# Moxibustion in Early Chinese Medicine and Its Relation to the Origin of Meridians: A Study on the Unearthed Literatures

**DOI:** 10.1155/2017/8242136

**Published:** 2017-02-19

**Authors:** Chang Huang, Jiankang Liang, Li Han, Juntian Liu, Mengyun Yu, Baixiao Zhao

**Affiliations:** ^1^Beijing University of Traditional Chinese Medicine, Beijing 100029, China; ^2^Beijing Huguosi Hospital of Traditional Chinese Medicine, Beijing 100029, China

## Abstract

Moxibustion is an integral part of Traditional Chinese Medicine (TCM). It achieved higher level of recognition and had more general application in ancient times than in contemporary life. As the vital historical sources, the records of unearthed literatures offered precious insights to Chinese social life pattern and medical practice in* Qin* and* Han* dynasties (221 BC–220 AD). There was no surprise that the bamboo and silk documents excavated from* Mawangdui* (马王堆) tomb,* Hantanpo* (*旱滩坡*) tomb, and other relics had a large amount of texts relevant to moxibustion. This research sorted moxibustion recordings from seven unearthed literatures and discovered that moxibustion had been developed into different modalities and utilized to treat many diseases at that time. In addition, the indications, contraindications of moxibustion, and the method of postmoxibustion care were also discussed. On this basis, some hints were provided to support the hypothesis that the practice of moxibustion led to the discovery of meridians. All our preliminary results in the research have drawn attention for this old therapy and given a new source for its application in clinic and scientific research.

## 1. Introduction

Moxibustion is an external therapy using burning moxa stick or cone to produce a warm sensation and moxa smoke on the acupoints [1]. It was regarded as a principal treatment in ancient China. The studies of the earliest known form of Chinese writing, Oracle Bone Script (*Jiagu Wen*, 甲骨文), indicated that moxibustion might have been applied in* Yin* dynasty (1600 BC–1046 BC) [2]. However, it is not as popular as acupuncture today, and* Zhenjiu* (*针灸*), acupuncture-moxibustion therapy, is often misconstrued as a medical practice that only uses needles to cope with diseases.

The meridians (*Jingmai*, 经脉) are core concepts for the practice of acupuncture-moxibustion and were first described in* The Yellow Emperor's Classic of Internal Medicine* (*Huangdi Neijing*, 黄帝内经). This theory was based upon earlier medical treatises that referred to the vessels (*Mai*, 脉). Many scholars agree that these earlier descriptions of the vessels influenced the development of the meridians. Modern Chinese researchers have worked for over 50 years on how the ancients discovered the vessels and gradually built the meridian theory, but without a definite conclusion [3]. The purpose of the current paper is to explore how the treatment method employed for the vessels, moxibustion, might have contributed to the evolution of the vessels to the meridians and thus to the development of meridian theory.

In China, bamboo and silk were the primary writing instruments before the widespread use of paper [4]. Since the beginning of the 20th century, a large number of bamboo slips and silk books have been unearthed from tombs of* Qin* (221 BC–206 BC) and* Han* (202 BC–220 AD) dynasties in China [5, 6]. They documented valuable medical materials and partially illustrated how Chinese medicine originated [7].

The study on the bamboo slips and silk books contributed to understanding the development of moxibustion before* Qin* and* Han* dynasties and offers a new source for its application in clinic and scientific research. Furthermore, the exploration of the relevance between moxibustion and vessels could give an additional insight into the nature or basis of the meridians. Hence, our article commences with a summary of the textual contents that discussed moxibustion from the unearthed literatures. There were a total of seven bamboo slips and silk books used in this research. From these we explored the practice of moxibustion in early China from such aspects as postmoxibustion care, indications, and different modalities of moxibustion by studying these records. After illustrating the circumstances of moxibustion's application at that time, some clues were offered to reflect the influence of moxa practice on the development of the meridians.

## 2. The Records of Moxibustion in the Unearthed Literatures

There are 25 bamboo slips and silk books involved with medicine among all unearthed literatures. Seven of them are closely related to moxibustion and they were listed in (Table 1).

*Cauterization Canon of the Eleven Vessels of the Foot and Forearm* and* Cauterization Canon of the Eleven Yin and Yang Vessels* excavated from* Mawangdui* tomb 3 (burial dated 168 BC) in* Changsha* of* Hunan* province are the earliest monographs covering not only vessels but also the application of moxibustion. The books presented the information of eleven vessels, a precursor of the twelve standard meridians. The vessel's names, trajectories, diseases generated from disharmony along those vessels, and therapeutic methods were set out. The majority of diseases mentioned in them were urological or pain-related and moxibustion was the only therapy [8]. As compared with* Cauterization Canon of the Eleven Vessels of the Foot and Forearm*,* Cauterization Canon of the Eleven Yin and Yang Vessels* discussed more diseases of vessels and classified them into two categories: disease that occurred when vessel perturbed (*Shidongbing*, 是动病) and disease that occurred when giving rise to vessel (*Suoshengbing*, 所生病) [9], so we inferred that its written time might be earlier. In the earlier book,* Cauterization Canon of the Eleven Vessels of the Foot and Forearm*, the sentence “it should be treated by moxibustion on a certain vessel” occurred at the end of introduction of each vessel. The explanation of this sentence was that the disease should be treated by moxibustion on the vessels that contributed to the onset of disease. Although the sentence was removed in* Cauterization Canon of the Eleven Yin and Yang Vessels*, the content of the postmoxibustion care was added.

*Model of the Vessels* is the book that mainly discussed the* Qi* (气) and vessels.* Qi* is usually described as the flow of energy around the body. The disrupted, blocked, or unbalanced* Qi* movement would lead to the generation of diseases. The book put forward the principle “taking the excess to fill up the deficiency” to treat diseases by moxibustion and* Bian* stones. The core idea contained in this principle is the concept “Balance,” which became an important part of TCM theory later. The book regarded that* Qi* moved in accordance with a certain rule and it would be beneficial when it arrived at lower body but harmful at the upper body. The disease would arise when* Qi* ran along the vessel and gathered at the upper body, and then moxibustion should be employed to treat it. Also, as the disease got worse, the stimulating intensity should be increased by moxibustion on another area that was above the previous treating area. Another significant part in this book was the discussion of moxibustion contraindication by a case study on treating carbuncle. The development of carbuncle was usually divided into incipient and later period. The main symptoms of the former one were redness, swelling, and ache, but without pyosis. When carbuncle continued to enlarge and the pus came into being at later period, moxibustion use was banned while flint and needle should be used to incise and drain [8].

*Book of the Vessels*, which was excavated in* Zhangjiashan* (张家山), is a collection of* Cauterization Canon of the Eleven Vessels of the Foot and Forearm*,* Cauterization Canon of the Eleven Yin and Yang Vessels*, and* Model of the Vessels* [10]. Therefore, its contents about moxibustion could be the duplication of these three books.

*Recipes for Fifty-Two Ailments* is the earliest manuscript of formulaology and moxibustion prescription. It contains about 14,700 words (maximum number of words in all silk books from* Mawangdui* tomb), 103 diseases, 283 formulas, and eight moxibustion prescriptions [11]. Some scholars proposed that there were eleven moxibustion prescriptions recorded in this silk book, but soon afterwards three prescriptions were found to be erroneously categorized to moxibustion for misprinting the word “炙” (*Zhi*, roast) as “灸” (*Jiu*, moxibustion) [12]. Thus Table 2 summarized eight moxibustion prescriptions in* Recipes for Fifty-Two Ailments*.

*Recipes for Fifty-Two Ailments* recorded moxibustion with different materials such as moxa, hessian, phaeodon, and* Scirpoides holoschoenus* in treating diverse diseases. The second prescription in “venomous snake bite” took advantage of white mustard seed to stimulate skin to blister, which was known as the earliest natural moxibustion. Nowadays, the famous “Sanfu moxibustion” (*Sanfu Jiu*, 三*伏灸*) which is primarily applied to cure winter diseases in summer has been the inheritance of this method. Fume moxibustion in the first prescription of “peritus ani,” meant combusting moxa and other herbs to produce smoke and heat on the affected part. In order to apply fume moxibustion conveniently, modern Chinese doctors have developed a sitting-moxibustion apparatus instead of traditional manual method in treating anus diseases [13].

*Recipes for Fifty-Two Ailments* also recorded the characters concerned with postmoxibustion care. The eighteenth prescription in chapter “scrotal hernia” proposed a caution that patients should avoid the invasion of exogenous pathogenic wind after moxibustion [14]. Postmoxibustion sore appeared frequently in moxibustion therapy. Ancients believed that it is necessary to treat the sore appropriately to prevent further deterioration, although it tightly coupled with the therapeutic effect [15]. Thereby, the method using Chinese medicinal herbs to treat moxibustion sore was also introduced in the second prescription in chapter “post-traumatic leg.”

*The Ultimate Principles in the Universe* is a sexual guide literature, in which the health care methods based on Taoist sexual practices were elaborated and moxibustion was a therapy to treat the diseases resulting from improper intercourse. “Seven impairments and eight supplements” (*Qisun Bayi*, 七损八益), a renowned guiding principle for sexual intercourse mentioned in the book, presented seven detrimental behaviors and eight beneficial behaviors for health [16]. People would be abnormally sweaty, wheezy, and vexed if they failed to follow these guiding principles in sexual activity. Without immediate treatment, these symptoms would get worse and induce endogenous heat (*Neire*, 内热), pathogenic factors that would attack the body in TCM. At this point, Chinese herbs or moxibustion should be taken to treat this disorder by regulating* Qi* [8].

*Wuwei Medical Slips*, unearthed from the* Hantanpo* tomb built in early Eastern* Han* dynasty (25 AD–220 AD), have listed 45 prescriptions and more than 100 herbs on the 92 bamboo slips and wooden tablets, which were made by pine and poplar wood. The application rules of moxibustion are the important contents in these slips. On the one hand, it was stated that certain parts of the body were not suitable for application of moxibustion at certain ages. For example, the heart was forbidden to carry out moxibustion on at age one, abdomen at age two, and back at age three [14]. As demonology influenced Chinese medicine quite a lot in ancient time, above-mentioned issue might originate from demonological therapies and could not be validated by scientific and clinical studies. Thus some of these opinions were not appropriate for applying in treatment now [17, 18]. On the other hand the treatment timing of moxibustion was mentioned. The ancients believed it was a critical factor to enhance efficacy that moxibustion should be applied on the different acupoints in accordance with different time. Such a record is an embryonic form of “midnight-midday ebb-flow” (*Ziwu Liuzhu*, 子午流注), which is built on the basis of biorhythm by the medical practitioners in the successive dynasties. In addition, a treatment protocol for postmoxibustion care was also provided in the slips. The method was to boil smashed* Aconitum carmichaelii*,* Capsicum annuum*, and dry* Angelica* together with Bactrian camel milk and then apply them to the surface of sore [14].

## 3. The Practice of Moxibustion in Early China

Moxibustion was one of the oldest therapies for its invented time might trace back to primitive society. Mastering fire-making technique provided a prerequisite for moxibustion to take its shape. In cooking a meal or getting warm by using fire [19], people unexpectedly found that stimulating body's specific location could alleviate pain and suffering. Ancients summed up the regular stimulating methods and developed them into a new therapy. The scenario of doing moxibustion depicted on the oracle bones demonstrated that moxibustion had occurred in* Yin* dynasty [2]. Then it prevailed and became the mainstay of therapy during* Qin* and* Han* dynasties. Apart from unearthed literatures, the hand-down literatures (the literatures which were handed down through arrangement and transcription by generations of scholars) also indicated that moxibustion had been widely used in medical field and became a formal therapy at that time. The* Records of the Grand Historian* (*Shi Ji*, 史记), an official Chinese history book with a great level of influence, has covered 3000 years of history from* Yellow Emperor* to* Emperor Wu of Han*. It documented two moxibustion recipes in the biography chapter of* Cang gong*, whose medical cases were considered the earliest medical history records. Moreover, the moxibustion physician, as an ancient's profession, also frequently appeared in poetry of* Tang* (618 AD–907 AD) and* Song* (960 AD–1279 AD) dynasties [20].

When analyzing the reasons for the prevailing of moxibustion, three plausible explanations could be discovered. First, with the limited production technology, the craftsmanship of needles was in primitive stage and a majority of them were made of stone. The patients suffered from bloodletting, incision, and drainage by using* Bian* stones. By comparison, moxibustion was easy to be accepted for less suffering by patients. Second, the flammable materials for moxibustion with a wide range items were apt to search. The materials such as mugwort leaf,* Scirpoides holoschoenus*, and mulberry and peach tree branches could be used. Moxa floss, which is processed by dried leaves of* Artemisia argyi* (an easily cultivated herbaceous perennial plant), is regarded more appropriate than any other combustion materials for moxibustion currently [15]. Third, moxibustion displayed certain effect on some unapproved indications of acupuncture.* The Yellow Emperor's Classic of Internal Medicine* (*Huangdi Neijing*, 黄帝内经) said, “a disease that may not be treated by acupuncture may be treated by moxibustion.”

As for the indications of moxibustion, which are associated with the prevalent diseases in different eras, they are always of concern to scholars. During the periods of* Warring States* (475 BC–221 BC),* Qin* dynasty and* Han* dynasty, war was frequent and iron weapon appeared. Many soldiers were injured and died from continual warfare. Agriculture and handicraft, yet, largely developed; the use of lacquer-ware permeated into every walk of life and people suffered from paints allergy and rhus dermatitis. Hence, the diseases treated by moxibustion documented in the unearthed books were mainly affiliated to surgery, including traumatism, animal bites, purulence, and urinary and anorectal diseases [21, 22]. A part of the above still continues today in contemporary medical treatment. With advanced moxibustion therapy, the indications of moxibustion have been expanded. Over 20 years, experts have investigated moxibustion treatment of 364 diseases with animal experiment and human trials [23].

Heat syndrome (*Rezheng*, 热证) was referred to as full heat syndrome (*Shi Rezheng*, 实热证) and empty heat syndrome (*Xu Rezheng*, 虚热证). The former one with the symptoms such as red face, red eyes, a red tongue with yellow coating, and full rapid pulse was caused by the excess of* Yang* (阳) in the body or invasion by an external pathogenic factor. The empty heat is caused by a deficiency of* Yin* (阴) rather than an excess of* Yang* and usually has the symptoms of dry mouth, dry throat at night, night sweats, a peeled tongue, and a floating and rapid pulse. Whether the heat syndrome pertains to the indications of moxibustion is a controversial subject [24]. In the late Eastern* Han* dynasty,* Zhang Zhongjing* first put forward the idea that “moxibustion is not appropriate for heat syndrome” since the heat could make the fire hurt veins [25]. But scholars disagree with it based on the studies of unearthed literatures. The records in* Cauterization Canon of the Eleven Vessels of the Foot and Forearm* and* Recipes for Fifty-Two Ailments* indicated that some diseases that belong to heat syndrome should be treated by moxibustion. The* Yellow Emperor's Classic of Internal Medicine* also had the theory of “removing the stagnation of fire by heat” (*Yi Re Yin Re*, 以热引热). Moreover, the results of many clinical researches supported the idea that “heat syndrome could be treated by moxibustion” [26, 27], and the functions such as antipyretic, anti-inflammation, antiviral, and regulating immunity have been found to contribute to this therapeutic effect of moxibustion [15, 28, 29].

In terms of different materials and operating processes, various modalities of moxibustion have been developed in early China. By studying the earliest moxibustion monographs, it could be learned that different diseases occurring at the same vessel were treated with moxibustion in the same way. The operating method of moxibustion was relatively simple when it was incipiently practiced. With the compiling of* Recipes for Fifty-Two Ailments*, moxibustion was enriched and various modalities such as fume moxibustion, direct moxibustion, and natural moxibustion were exploited for different diseases. A range of combustible materials like tow or* Scirpoides holoschoenus* was mixed with moxa cone to make a fire for moxibustion. Another similar method of adding several materials to the moxa stick was developed into thunder-fire moxibustion (one of the moxibustion modalities that used moxa stick involved diverse herbs with different properties to serve different treatment purposes) during* Ming* dynasty (1368 AD–1662 AD) [30]. Although moxa stick moxibustion was the most commonly utilized among all moxibustion modalities in recent years, it had not yet occurred in the* Qin* and* Han* dynasties. The earliest record about moxa stick moxibustion was in* Medical Secretes of an Official* (*Waitai Miyao*, 外台*秘要*) at* Tang* dynasty [31].

In order to achieve better therapeutic effect, ancient Chinese doctors realized the significance of postmoxibustion care early. The postmoxibustion care was mainly from two aspects of the treatment of moxibustion sore and the notice of daily activity after moxibustion. For one thing, most medical practitioners believed that the moxibustion sore issued from cautery was closely associated with the curative effect, so ancients generally preferred to use scarring-moxibustion and realized that cauterized sore after strong stimulation was inevitable [15]. A study indicated that the thought of Greek medicine paralleled Chinese medicine. In the middle ages of French and Greek, the common treatment named “cauterization” had a similar core idea with ancient scarring-moxibustion [32, 33]. Nevertheless, it was necessary to treat moxibustion sore for the infection of sore could be harmful to the patients' health. The records of* Recipes for Fifty-Two Ailments* and* Wuwei Medical Slips* were significant initiation at moxibustion sore treatment; then the later practitioners developed various methods for healing the postmoxibustion sore during a long-term medical practice. Additionally,* Cauterization Canon of the Eleven Vessels of the Foot and Forearm* and* Recipes for Fifty-Two Ailments* had put forward certain principles on diet, dressing, and exercise which contributed to promoting the effect of moxibustion. Although some of the clinical efficacy of these treatments should be further validated, the viewpoint of postmoxibustion care in ancient time still gave a lead to modern moxibustion therapy and offered valuable nursing experience after moxibustion for practitioners.

## 4. The Relationship between Moxibustion and the Origin of Meridians

Meridian theory is an important part of Chinese medicine; its origin and nature are still shrouded in mystery. The early development of vessels, a rudimentary model of meridians, is primarily in relation to moxibustion practice. In the* Cauterization Canon of the Eleven Vessels of the Foot and Forearm* and* Cauterization Canon of the Eleven Yin and Yang Vessels*, eleven vessels were presented in detail, but contemporary meridian theory that came out from* Yellow Emperor's Classic of Internal Medicine* has twelve meridians [34]. As the content of meridians in* Yellow Emperor's Classic of Internal Medicine* was influenced by the introduction of vessels in two silk books, the evolution of vessels to meridians could be interpreted by making a comparison between these three books (Table 3).

As the comparative study indicated, there were many differences between the descriptions of the vessels and meridians. Each of the eleven vessels has their starting point, terminal point, and trajectories, but no acupoints. The starting and terminal points on the vessels are all on the different area of body, while some of them on meridians are acupoints. The vessels are isolated from each other but the meridians are connected in a sequence with a cyclical circulation. The viscera (*Zangfu*, 脏腑) theory had not been built and the connections between viscera and vessels had also not been illustrated at the finishing time of two silk books; only some clues could be found from the relationship between diseases of vessels and viscera. For the treatment, at the beginning, moxibustion was applied on vessels to treat the diseases generated from disharmony of them. Later, acupuncture was discussed more in* Spiritual Pivot* (*Lingshu Jing*, *灵枢*经). In the* Cauterization Canon of the Eleven Vessels of the Foot and Forearm*, the Chinese glyphs of the word “vessel” was in proximity to another word “温” (*Wen*, warm) which was an adjective meant moderate temperature [35]. When treating with moxibustion, warm sensation will be applied on the surface of the skin. If the ancients realized the existence of vessel when doing moxibustion, they might name the vessel with a character that had close relationship with moxibustion according to the principle of creating characters [36]. This could explain why the earliest Chinese character of vessel was “
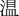
” (*Mai*, vessel) and then provide a hint that the realization of vessel may be connected with the application of moxibustion.

The phenomenon of propagated sensation along meridians means that people can sense distension, numbness, itching, tingling, or warmth run from the treating sites to distal end along the trajectory of meridian after the stimulation of acupuncture, moxibustion, or electrical stimulator [37]. Scholars believed the line of propagated sensation contributed to the description of trajectory of vessels by ancients [38, 39]. The records of treatment in the two earliest vessel books suggested that moxibustion could be the principal therapy at that time. So it might inspire someone to conjure up that the ancients had the specific sensation propagated along a certain route after using moxibustion. This propagated sensation is also called moxibustion-esthesia and it was recorded throughout the ancient literatures.* The Spiritual Pivot* stated that “when warm* Qi* travelled along the vessels and arrived around the body, then the blocked blood vessels will open up.”* Huangdi's Mingtang Moxibustion Classic* (*Huangdi Mingtang Jiujing*, 黄帝明*堂灸*经) was a treatise on moxibustion created in* Tang* dynasty and the author's name was not recorded. The theory presented in it was “when moxibustion on the inaccurate acupoints, the thermal power could not flow away to cure diseases.” Moreover,* Thousand Golden Prescriptions *(*Qianjing Yaofang*, 千金要方) described moxibustion-esthesia as the sensation of water running down when moxibustion was treated on the shoulder blades.

Likewise, modern scholars perceived the presence of moxibustion-esthesia and proved it by clinical research. In 2000, Chinese researchers enrolled a total of 829 patients to analyze their body's reaction after moxibustion. It was reported that 733 patients had the propagated sensation travelling along meridians [40]. Professor* Zhou Meisheng*, a distinguished moxibustion specialist who is the author of* Moxibustion Criterion* (*Jiusheng*, *灸绳*), believes that inducing moxibustion-esthesia is an important method to improve curative effect of moxibustion. He came up with the theory of* Three Stages of Moxibustion-esthesia*. The first stage is that the sensation of numbness, itching, tingling, or warmth would run along the meridian at the beginning of moxibustion. Secondly, the special sensation would arrive in the affected area and the intensity of sensation is correlated with diseases. The special sensation appearing in the affected area that represents the moxibustion is exerting the therapeutic action. Finally, special sensation may stop propagating or travel into the next meridian [41]. Based on this theory, some scholars developed a new method of moxibustion called* Thermal Sensitivity Moxibustion* [42, 43]. They suggested that some acupoints become sensitive when people are undergoing pathological conditions. Doctors should apply moxibustion on these acupoints to induce moxibustion-esthesia, because moxibustion at these heat-sensitive acupoints had a better efficacy than conventional moxibustion.

## 5. Conclusion

This study of bamboo slips and silk texts from two thousand years ago gives a preliminary description of the circumstances of medical practice and confirmed the important historical status of moxibustion during the* Qin* and* Han* dynasties. Moxibustion was used to treat various diseases and its indications contained almost all the common diseases when it appeared in these early texts. Throughout the records of moxibustion in unearthed texts we see that moxibustion developed from a unitary modality to multiple modalities. Different combustion materials and steps of the operation were chosen for different diseases, and some modalities were still applicable for treatment in modern life. Postmoxibustion care was usually neglected by modern people, while the ancients attached great importance to it and gave some advice not only for the treatment of moxibustion sore but also for the daily activity after moxibustion. This experience deserved to be further researched for its application at clinic in the future. We feel that there is little doubt that moxibustion has close links with the meridians. As the preceding discussion has illustrated, moxibustion was developed earlier than acupuncture and its application has a greater chance to give rise to the origin of vessels and the evolution of the vessels to meridians. The ancients might have perceived the propagated sensation along some trajectories after having moxibustion. After analyzing, certain laws were found in the trajectories and they came up with the concept of vessels. Then the eleven vessels were gradually developed into a relatively complete meridians system over a more extended period of practice.

The unearthed texts contain rare historical information, many of them have not been made public or were damaged from lack of proper preservation. This paper collected seven existing bamboo and silk texts with records of moxibustion. In addition, there may have been interpretative mistakes due to deviation in the analyses of these unearthed texts since Chinese written characters have undergone significant changes over time. As the new bamboo slips and silk texts are further arranged and made public by archaeologists and scholars, further research on them might provide additional insight into the origin of moxibustion and the meridians. But, as importantly, we feel this is a good chance to draw people's attention to the innovations of this conventional therapy.

## Figures and Tables

**Table 1:** The moxibustion-related unearthed literatures.

Book involved with moxibustion	Excavated time	Excavated sites	Inferential completion date	Material	Main content
*Cauterization Canon of the Eleven Vessels of the Foot and Forearm* (*Zubi Shiyi Maijiujing, *足臂十一脉灸经)	1973	*Mawangdui Han* tomb 3 (*Changsha*, *Hunan* province)	During late *Qin* and early *Han* dynasties	Silk book	Names, trajectories, and diseases of 11 vessels and moxibustion
*Cauterization Canon of the Eleven Yin and Yang Vessels* (*Yinyang Shiyi Maijiujing, *阴阳十一脉灸经)	1973	*Mawangdui Han* tomb 3 (*Changsha*, *Hunan* province)	During late *Qin* and early *Han* dynasties	Silk book	Name, trajectories, and diseases of vessels
*Model of the Vessels* (*Maifa, *脉法)	1973	*Mawangdui Han* tomb 3 (*Changsha*, *Hunan* province)	During late *Qin* and early *Han* dynasties	Silk book	(1) Relationship between* Qi *and vessels (2) Using moxibustion and *Bian* stone to treat diseases (3) Palpating the trajectories of vessels to diagnose diseases
*Recipes for Fifty-Two Ailments* (*Wushi'er Bingfang, *五十二病方)	1973	*Mawangdui Han* tomb 3 (*Changsha*, *Hunan* province)	During late *Qin* and early *Han* dynasties	Silk book	Recipes for treating 52 diseases in internal medicine, surgery, and pediatrics
*The Ultimate Principles in the Universe* (*Tianxia Zhidao Tan, *天下至*道谈*)	1973	*Mawangdui Han* tomb 3 (*Changsha*, *Hunan* province)	Before or during *Han* dynasty	Bamboo slips	Principles and skills of health care in sexual behavior
*Book of the Vessels* (*Maishu, *脉书)	1983-1984	*Zhangjiashan* Western *Han* tomb 247 (*Jiangling*, *Hubei* province)	Before or during Western *Han* dynasty	Bamboo slips	(1) Diseases in different parts of the human body and their symptoms (2) Trajectories of the vessels and their related diseases
*Wuwei Medical Slips* (*Wuwei Handai Yijian, 武威*汉代医简)	1972	*Hantanpo Han* tomb (*Wuwei*, *Gansu* province)	Before Eastern *Han* dynasty	Wooden slips and tablets	Recipes for treating diseases in internal medicine, surgery, gynecology, and andrology

**Table 2:** The moxibustion prescriptions in *Recipes for Fifty-Two Ailments*.

Disease	Serial number in each chapter	Modality	Material	Moxibustion method
Venomous snake bite	Number 2 prescription	Natural moxibustion	Mustard poultices	Putting mustard poultices on the patient's vertex cranii (GV 20)
Wart	Number 1 prescription	Sear moxibustion with moxa stick	*Scirpoides holoschoenus*	Lighting the rope of *Scirpoides holoschoenus* to cauterize the terminal of wart and then take it out
Dysuria	Number 6 prescription	Direct moxibustion	Unknown	Moxibustion on middle toe of right foot
Scrotal hernia	Number 10 prescription	Direct moxibustion	Mugwort leaves, tow	Burning tow wrapped with mugwort leaves on vertex cranii (GV 20) until moxibustion scar appeared
Scrotal hernia	Number 18 prescription	Moxibustion after stone needle therapy	Unknown	Moxibustion on the vulnus after stone needle cutting or “*Taiyin*” (太阴) and “*Taiyang*” (太阳)
Scrotal hernia	Number 23 prescription	Direct moxibustion	Unknown	Moxibustion on left shank (based on *A-B Classic of Acupuncture and Moxibustion*, the treatment points might be SP8, LR5, or KI8)
External hemorrhoid or anal fistula	Number 1 prescription	Sear moxibustion with moxa stick	Unknown	Taking moxibustion to cauterize the terminal of hemorrhoid and then twisting it off
Pruritus ani	Number 1 prescription	Fume moxibustion	Mugwort leaves, mushroom on the willow	Burning mugwort leaves and mushroom form the willow in a hole and applying moxibustion smoke to the patient's anus

**Table 3:** The comparison between *Yellow Emperor's Classic of Internal Medicine*, *Cauterization Canon of the Eleven Vessels of the Foot and Forearm,* and *Cauterization Canon of the Eleven Yin and Yang Vessels*.

Name of book	*Cauterization Canon of the Eleven Vessels of the Foot and Forearm*	*Cauterization Canon of the Eleven Yin and Yang Vessels*	*The Yellow Emperor's Classic of Internal Medicine*
Nomenclature	Foot/hand *Yin*/*Yang* meridians	*Yin*/*Yang* meridians or regional anatomy position meridians	Affiliated viscus foot/hand *Yin*/*Yang* meridians

Writing form of a character of meridian	“*Mai*” ( 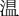 )	“*Mai*” (脉)	“*Jingmai*” (经脉)

Direction of meridians circulation	Centripetal	Shoulder meridian and hand* Taiyin *meridian: axofugal Others: centripetal	Hand *Yin* meridian: thorax to hand Hand *Yang* meridian: hand to head Foot *Yin *meridian: feet to thorax Foot *Yang *meridian: head to feet

Number of meridians	11	11	12

Relationship between meridians	No correlation	No correlation	Junction by head-tail in regular sequence

Amount of acupoints	None	None	160 points

Amount of diseases	78	*Shidongbing*: 60 Suoshengbing: 87	*Shidongbing*: 74 Suoshengbing: 143

Treatment for diseases	Moxibustion	Moxibustion	Acupuncture; moxibustion; decoction

Relations to viscera and bowels	Only two meridians connected with viscera and bowels	Only three meridians connected with viscera and bowels	12 meridians all have their own affiliated viscera and bowels
